# MG53′s new identity

**DOI:** 10.1186/2044-5040-3-25

**Published:** 2013-11-01

**Authors:** Jennifer R Levy, Kevin P Campbell, David J Glass

**Affiliations:** 1 Howard Hughes Medical Institute, Senator Paul D Wellstone Muscular Dystrophy Cooperative Research Center, The University of Iowa, Iowa City, IA 52252, USA; 2 Department of Molecular Physiology and Biophysics, The University of Iowa, Iowa City, IA 52252, USA; 3 Department of Neurology, The University of Iowa, Iowa City, IA 52252, USA; 4 Department of Internal Medicine, Roy J and Lucille A Carver College of Medicine, The University of Iowa, Iowa City, IA 52252, USA; 5 Novartis Institutes for Biomedical Research, 100 Technology Square, Cambridge, MA 02139, USA

**Keywords:** Mitsugumin 53, MG53, Insulin receptor, Insulin signaling, E3 ubiquitin ligase, Metabolic syndrome, TRIM72

## Abstract

Mitsugumin 53 (MG53) is a relatively newly identified tripartite motif-containing (TRIM) family muscle-specific E3 ubiquitin ligase that is expressed in skeletal muscle and the heart. It has been postulated to facilitate repair by targeting the site of an injury, and acting as a scaffold for assembly of a repair complex made up of dysferlin, annexin V, caveolin-3, and polymerase I and transcript release factor (PTRF). A recent letter published in *Nature* by Song *et al.* proposes an alternate function for MG53: as an E3 ligase that targets the insulin receptor and insulin receptor substrate 1 (IRS1) for degradation, therefore regulating muscle insulin signaling. This work is exciting, as it not only presents a novel role for MG53, but also suggests that muscle insulin signaling has a systemic influence on insulin resistance and the metabolic syndrome.

## Background

Metabolic syndrome is a disorder of increasing prevalence, especially in developed countries; it is associated with an increase in the incidence of obesity and the often-associated type 2 diabetes. It is defined as a cluster of conditions comprising hypertension, high blood glucose levels, abnormal cholesterol levels and central obesity that, when they occur in combination, increase the risk for cardiovascular disease and type 2 diabetes [1]. The majority of insulin-stimulated glucose disposal occurs in skeletal muscle [2,3], and therefore it is likely that insulin resistance in skeletal muscle is a precursor to systemic metabolic dysfunction.

Mitsugumin 53 (MG53) is a cardiac and skeletal muscle-specific protein, which was identified in a screen using an immuno-proteomic monoclonal antibody library against proteins present in striated muscle [4]. MG53, also known as tripartite motif 72 (TRIM72), has a molecular weight of 53 kD, and contains the prototypical tripartite motif, which includes a RING, B-box and coiled-coil moiety. MG53 also has a SPRY domain at the carboxy terminus [5]. Although MG53 contains a canonical E3 ligase RING finger domain, its E3 ligase activity was not confirmed until recently [6].

Cai *et al*. generated an MG53-null mouse, which developed slowly progressive muscle pathology: mice aged 10 to 11 months had increased numbers of central nuclei and decreased muscle fiber diameter. The skeletal muscle from MG53-null mice had increased Evans blue dye uptake after downhill running, suggesting that these mice have increased susceptibility to membrane injury. Further experiments by this group of researchers implicated MG53 as a central player in the repair of membrane damage; it acts by binding to phosphatidylserine at the damage site and acts as a scaffold to recruit a complex of repair proteins that includes dysferlin, annexin V, caveolin-3 and polymerase I and transcript release factor (PTRF) [5,7]. Although MG53 has been found to be upregulated in muscular dystrophy patients [8], no pathogenic MG53 mutations have been identified.

## Discussion

Recently, Song *et al.* published a series of experiments that identify a novel role for MG53 as a mediator of insulin signaling [6]. The authors observed that animal models for metabolic syndrome (high-fat diet (HFD)-induced obese mice, *db/db* diabetic mice, spontaneously hypertensive rats and non-human primate models of metabolic syndrome), as well as obese humans, have increased expression of MG53 compared to control animals and non-obese humans.

To determine if MG53 is required for pathogenesis of metabolic syndrome, the authors fed MG53-null mice and wild-type littermates a HFD. MG53-null mice were protected from increases in body weight, blood pressure, and serum cholesterol and triglyceride levels. Further, the loss of MG53 blocked HFD-induced systemic insulin resistance. Conversely, a transgenic mouse model that overexpresses MG53 had the hallmarks of metabolic syndrome (increased body weight, blood pressure and serum cholesterol levels) and insulin resistance in the absence of a HFD [6].

The authors delved into the mechanism for the regulation of insulin signaling by MG53. Insulin stimulation leads to autophosphorylation of the insulin receptor. This leads to phosphorylation of insulin receptor substrate 1 (IRS1) and insulin receptor substrate 2 (IRS2), which in turn activates the phosphatidylinositol-3-OH kinase (PI(3)K)-Akt-GSK3-beta signaling pathway. This pathway mediates glucose homeostasis in skeletal muscle [9]. Previously, Ko and colleagues [10] showed that MG53 overexpression prevented IRS1 phosphorylation and myogenesis in C2C12 myoblasts. The current study found that MG53-null tissues had increased insulin signaling, whereas MG53 overexpression suppresses insulin signaling. Changes in insulin signaling accompanied marked changes in insulin receptor and IRS1 protein levels, but not mRNA levels. Surprisingly, skeletal muscle insulin resistance in MG53 transgenic mice preceded whole-body insulin disorders (obesity and multi-organ insulin resistance). Specific inhibition of the E3 ubiquitin ligase activity of MG53 by deletion of the RING finger domain or by mutation of a cysteine to an alanine at position 14 suppresses the effects of MG53 on insulin receptor activity and ubiquitination. This supports a model for MG53 targeting insulin receptor degradation via its E3 ubiquitination ligase activity; the downstream ubiquitination of IRS1 seems dependent on the initial insulin receptor degradation, since IRS1 was not degraded upon IGF1 stimulation. Therefore it seems that IRS1 may be a 'bystander’ – ubiquitinated due to its proximity to the insulin receptor/MG53 complex. The negative regulation of insulin signaling in skeletal muscle by MG53 triggers systemic changes in insulin function and metabolism.

## Conclusions

The compelling results presented in Song *et al.* suggest a novel role for MG53. It is interesting to speculate on how to reconcile its new identity as an E3 ubiquitin ligase that targets insulin signaling pathways with its canonical role as a membrane repair protein. It is possible that MG53 performs two disparate functions in skeletal muscle – it could exist in two functional pools, perhaps mediated by localization or post-translational modification. Alternatively, its roles may be synergistic. For instance, the direct interaction of MG53 with the insulin receptor and IRS1 [6] could direct MG53 to the cell surface, where it will be readily available when sarcolemmal disruptions require its activity as a repair protein. In support of a correlation between muscle membrane repair defects and insulin resistance, myocytes from *db/db* diabetic mice show increased dye uptake after laser damage, indicating reduced efficiency of membrane repair. Downhill running of *db/db* mice showed increased permeability of myocytes from these mice to Evans blue dye compared to wild-type mice [11]. Interestingly, deletion of the MG53-interacting proteins PTRF or caveolin-3 cause glucose intolerance and insulin resistance, as opposed to the protective effect seen in MG53-null mice, suggesting that members of the membrane repair complex have opposing roles in insulin signaling [12,13].

Alternatively, the E3 ubiquitin ligase activity of MG53 may act on targets in addition to insulin receptors and IRS1. The ubiquitin-mediated degradation of these potential targets may facilitate membrane repair using a mechanism that is not yet known. Interestingly, MG53 has recently been shown to interact with and attenuate the activity of sarcoplasmic reticulum calcium-ATPase 1a (SERCA1a) [14] – it is possible that SERCA is an additional target for the E3 ubiquitin-ligase activity of MG53.

One of the more surprising findings by Song *et al*. is that the insulin resistance observed in MG53 transgenic mice preceded changes in body weight and composition, suggesting that MG53 action in muscle alters the whole-body energy balance. The effects of insulin signaling specifically in muscle have been studied previously in mice with muscle-specific inactivation of the insulin receptor gene (muscle-specific insulin receptor knockout (MIRKO) mice) [15]. These mice show relatively minor changes in glucose homeostasis [15], possibly because the redistribution of substrates to adipose tissue causes increased adiposity in these mice [16]. It is possible that Song *et al*. observed a similar phenomenon in the MG53-transgenic mice: overexpression of MG53 targets insulin receptors for degradation, which could lead to redistribution of substrates to adipose tissue and, subsequently, metabolic syndrome.

Song *et al*. found elevated levels of the MG53 protein in obese humans and in several animal models for insulin resistance and metabolic disorders, including HFD-induced obese mice. It is unclear what mechanism is responsible for increased MG53 expression in situations of nutritional overload. Interestingly, a recent study by Ko and colleagues [17] confirms that MG53 is a ubiquitin E3 ligase that induces IRS-1 ubiquitination, and that MG53-null mice fed a high-fat and high-sucrose diet are protected from insulin resistance. However, this group found that muscle from HFD-fed mice and type 2 diabetes patients had normal levels of the MG53 protein. Ko and colleagues also challenged the finding by Song *et al*. that insulin receptor is a substrate of MG53 because insulin receptor protein levels and phosphorylation are unchanged when MG53 is overexpressed in myoblasts or genetically disrupted in knockout mice. These inconsistencies highlight a need for further study of the role of MG53 in insulin signaling and metabolism.

Recently, Weisleder *et al.* explored recombinant MG53 protein therapy in mouse models of muscular dystrophy [18]. They found that exogenous recombinant human MG53 reduced muscle membrane damage and decreased muscle pathology in *mdx* mice. While these results suggest MG53 protein administration could be therapeutic for Duchenne and potentially other muscular dystrophies, the results from Song *et al.* should serve to caution researchers to examine insulin resistance and other hallmarks of metabolic disorders in treatment groups. It is even possible that changes in insulin signaling are somehow driving the improved pathology observed in MG53-treated *mdx* mice.

In addition to discovering a new cellular role for MG53, Song *et al.* identified MG53-mediated suppression of insulin signaling as having the capacity to underlie systemic insulin resistance and metabolic syndrome. Muscle researchers should be excited to consider MG53 and skeletal muscle insulin signaling as central players in the development of metabolic syndrome.

## Abbreviations

HFD: High-fat diet; IRS: Insulin receptor substrate; MG: Mitsugumin; MIRKO: Muscle-specific insulin receptor knockout; PI(3)K: Phosphatidylinositol-3-OH kinase; PTRF: Polymerase I and transcript release factor; SERCA: Sarcoplasmic reticulum calcium-ATPase; TRIM: Tripartite motif.

## Competing interests

DJG is an employee of Novartis.

## Authors’ contributions

JRL, KPC and DJG wrote the commentary. All authors read and approved the final manuscript.
